# Centering ability and dentin removal of rotary systems in curved root canals

**Published:** 2009-07-06

**Authors:** Saeed Moradi, Ali Talati, Ali Monajem Zadeh

**Affiliations:** 1 *Department of Endodontics, Dental School/Dental Research Center, Mashad University of Medical Sciences, Mashad, Iran.*; 2 *Endodontist, Mashad, Iran.*

**Keywords:** Centering ability, Medin, Mtwo, Race, Root canal, Rotary instruments

## Abstract

**INTRODUCTION:** The aim of this study was to compare centering ability and dentin removal of three rotary systems in curved root canals of extracted teeth.

**MATERIALS AND METHODS:** Sixty root canals of mandibular first molars with curvatures ranging between 25-35^o^ were divided into three groups of 20 teeth each. Based on pre-instrumentation radiographs that assessed the angle and the radius of canal curvatures, teeth with curvatures were equally spread between the three groups. The root canals were sectioned horizontally at two levels before preparation and then remounted onto the muffle. All root canals were prepared using a low-torque control motor with Mtwo or Medin or Race instruments. Cross sectional images were obtained before and after instrumentation. Cross-sectional area and centering ability were evaluated. The data were analyzed using the one-way ANOVA and Tukey tests.

**RESULTS:** Neither instrument fracture nor permanent deformation occurred during preparations. The best centering ability was obtained by Mtwo instruments compare to Race and Medin instruments. In the coronal and middle sections, Mtwo removed less dentin than Race and Medin; while the difference in the apical section was not significant.

**CONCLUSION:** Under the conditions of this study, the debridement of root canals was more conservative with Mtwo. The canals prepared with these instruments were better centered in all three regions of the root.

## INTRODUCTION

Success of root canal treatment greatly depends on the complete removal of microorganisms and necrotic tissue through chemo-mechanical preparation of root canal system which includes debridement of infected dentin and organic tissue (1). The aim of instrumentation is preparing a continuously tapered root canal area with increasing apico-coronal diameter which facilitate the irrigation and handling of the instruments (2,3). Effective root canal preparation should also provide a three dimensional area for proper condensation of root filling materials to establish an acceptable apical seal. Anatomic limitations of root canals such as curvatures make debridement difficult. Many instruments have been presented to overcome these problems, but only a few seem to be capable of obtaining the primary objectives of root canal preparation (4,5).

It has been shown that root canal preparation using rotary nickel-titanium instruments facilitates root canal shaping and also maintains the canal curvature, even within severely curved canals (5-8). Many manufacturers have introduced new instruments with various novel designs and claims of superb preparation and quality.

There are many variations in the design of NiTi rotary instruments and accordingly, many investigations have assessed their quality (1,4,8).

Recently introduced rotary instruments include Medin, Mtwo and Race which are used world-wide. Investigations that assess these instruments are rare; therefore we aimed to compare the root canal shaping ability of recently introduced rotary instruments. Cross-sectional area and centering ability were the two parameters evaluated.

**Table 1:** The degree of canal curvature in study groups before canal instrumentation

Groups	n	Mean	SD
Mtwo	**20**	**28.9000**	**3.20197**
Race	**20**	**28.9400**	**3.20385**
Medin	**20**	**30.4250**	**3.32148**
Total	**60**	**29.4217**	**3.93779**
Results	**P= 0.546**	**F= 0.611**	

## MATERIALS AND METHODS

Sixty freshly extracted human permanent mandibular first molars were selected for this study. The inclusive criteria wereas follows: 1) mesiobuccal canal curvature between 25- 35 degrees and the radius between 4-9 mm; 2) mature and intact apices; and 3) apical diameter of canals compatible with file size #10. Coronal access cavities were prepared using diamond burs (FG Swiss, Tec Swiss) and the apical patency of root canals was confirmed with k-file (Dentsply Maillefer, Ballaigues, Switzerland) size #10.

Standardized radiographs were taken prior to instrumentation with the (Dentsply Maillefer, Ballaigues, Switzerland) file size #10 has been inserted into the mesiobuccal canal in order to determine the degree and radius of the curvature.

The X-ray tube (Siemens, Heliodent, Germany) was aligned perpendicular to the root canal. The exposure time (0.125; 70Kv, 7mA) was the same for all radiographs. The degree and radius of canal curvature were obtained from these preoperative radiographs with a computer program Image Pro plus 5.0 (Media cybernetics, silver spring MD, USA). The degree of curvatures was determined according to Schneider method (9) and the radius of the curvature was determined according to Schafer method (10). The teeth were divided into three groups of 20 each. The homogeneity of the groups with respect to the degree and the radius of curvature were evaluated using analysis of variance (ANOVA) and post-hoc student-Newman-Keuls test (Tables 1 and 2).

All teeth were shortened to 12 mm by decoronation. A modified Bramante muffle system (11) was used to assess the criteria. The specimens were embedded in acrylic resin in a muffle, which was specially prepared for this study. The inner surfaces of the muffle had grooves which made traces on the surface of the acrylic resin. These traces served as a guide to help to reassemble the pieces of the sectioned blocks. The blocks were sectioned horizontally by a thin cutting disk (0.3-mm thick) at two levels: one 4 mm from the apex and the other 8 mm from the apex. The disk was mounted on a special machine (Zooeek Z600, Germany) for cutting the blocks.

Photographs were taken of all three cross-sections of each tooth under a stereomicroscope connected to a charge coupled device (CCD) camera (Nikon digital sight Ds-U1, Tokyo, Japan) at a fixed position. Magnification was achieved using a special mounting device that enabled exact repositioning of the sections. The sections were reassembled in the muffle.

Group 1 was assigned for preparation with Mtwo (VDW, Munich, Germany) instruments, group 2 with RaCe instruments (FKG Dentaire Company, Switzerland), and group 3 with Medin (MEDIN co, Czech) instruments. The working length for all canals was assigned 0.5 mm from the length at which the tip of a size l0 file could be visualized at the apical foramen when viewed under a stereomicroscope (Nikon sm Z1000, Tokyo, Japan).

All rotary instruments in this study were set into permanent rotation with a 4:1 reduction handpiece (WD-66 EM: W&H. Buermoos, Austria) powered by a torque limited electric motor (Endo IT motor: VDW). For each file the individual torque limit and rotational speed used were carried out according to manufacturer's instructions.

In all canals irrigation was performed after each file change with 0.2 mL of a 5.25% NaOCl solution followed by 2 mL of a 17% EDTA solution and a final rinse with 2 mL saline.

After instrumentation, all sectioned canals were separated, and then photographed in the same manner as pre-treatment photographs.

**Table 2:** The radius of canal curvatures in study groups before canal instrumentation

Groups	n	Mean	SD
Mtwo	**20**	**6.1950**	**1.51187**
Race	**20**	**6.2300**	**1.48237**
Medin	**20**	**6.2100**	**1.50223**
Total	**60**	**6.2117**	**1.47569**
Results	**P= 0.997**	**F=0.003**	

**Table 3:** Centering ability of coronal section of study groups

Groups	n	Mean	SD
Mtwo	**20**	**0.9600**	**0.04963**
Race	**20**	**0.8905**	**0.07837**
Medin	**20**	**0.9020**	**0.07245**
Total	**60**	**0.9175**	**0.07350**
Results	**P= 0.004**	**F=6.010**	

The shaping ability of the rotary instruments was evaluated using the computer program IMAGE PRO plus 5.0 as follows:

1- Cross-sectional area: cross-sectional surface area of each section was measured both before and after instrumentation.

2- Centering ability: centering ability of the instruments towards the original canal was evaluated by the ratio of X_1_-X_2_/Y_1_-Y_2_ according to the method developed by Garip (12); in this formula X_1_ and Y_1_ represent the thickness of the internal and external sides of the canal wall, respectively, mesiodistally, before instrumentation. A result with ratio 1 indicates that the canal has remained centered and a result less than 1 indicates deviation of the canal outward, and result of more than one show that the canal deviates inward.

Mesiodistal deviation of curvature was studied as most changes after instrumentation occur in this aspect. Data were analyzed with one-way ANOVA and Tukey tests at a significant level of 0.05 using SPSS 11.0 (SPSS Inc., Chicago, IL, USA).

## RESULTS

After instrumentation of root canals no instrument fracture or permanent deformation were observed.

The mean ratios of all sections indicated that the centering ability of Mtwo instruments was significantly greater than others (P<0.05); while this difference was not significant between Race and Medin Tables 3-5.

**Table 4:** Centering ability of middle section of study groups

Groups	n	Mean	SD
Mtwo	**20**	**0.9185**	**0.06184**
Race	**20**	**0.7450**	**0.05206**
Medin	**20**	**0.7545**	**0.05226**
Total	**60**	**0.8060**	**0.09713**
Results	**P= 0.000**	**F=61.612**	

**Table 5:** Centering ability of apical section of study groups

Groups	n	Mean	SD
Mtwo	**20**	**0.9060**	**0.06012**
Race	**20**	**0.6120**	**0.03238**
Medin	**20**	**0.6130**	**0.03230**
Total	**60**	**0.7103**	**0.01459**
Results	**P= 0.000**	**F=301.922**	

There was no statistical difference between groups when comparing pre-instrumentation section areas. However, after instrumentation there was difference between the three groups; in Mtwo group, canals had the smallest coronal and middle cross section areas compared to the two other instruments (P<0.05) indicating less dentine removal in these sites. No statistical significant difference was observed between Race and Medin groups in these two regions after instrumentation.

The results also showed no statistically significant difference in apical cross-section in all groups after instrumentation.

## DISCUSSION

Canal preparation involves elimination of necrotic tissues and debris, and shaping of root canals. It is fundamental that root canal preparation should not change the primary shape of the canal (13).

Instruments that can follow the path of the canal and are able to remain centered in the canal, are good choices for root canal preparation (14,15). Many studies have shown better efficacy of rotary instruments in comparison with hand instruments, here, the comparison of three rotary instruments was considered (16,17).

In this study torque limited electric motor [Endo ZT motor: (VDW)] was used for instrumentation. This electric motor also controlled the speed and torque of the instruments. Endo IT electric motor can be programmed for different types of rotary instruments and is able to rotate the file in reverse direction when the file is locked in canal in order to prevent file separation. Also this electric motor produced better results when compared with the others (18).

In this study, and Yang *et al.*’s (19), serial sectioning method and special muffle were used in order to evaluate centering ability and the amount of dentin removal. Al-Omari (20) and Yun (21) used simulated canals in their studies. As physical and chemical characteristics of these acrylic canals differ from natural tooth, an advantage of the study was the fact that it did utilize natural extracted teeth. In addition the root canal curvature, the radius was measured according to determined ranges, and with the purpose of achieving precise measurements.

Race files have a single triangular cross-sectioned shape with alternating cutting edges. Schafer and Vlassis (22) showed that race created no canal aberration and maintained working length well in curved canals. Other authors showed more canal transportation with race files, compared with profile and K3 (23) and with Hero shaper and Protaper (15).

The Mtwo cross-sectional design resembles S-shaped file with two blades; it also has a positive rake angle. The use of Mtwo resulted in the lowest canal transportation and the best centered canal preparations in this study and in Yun and Kims (21).

As results show, Mtwo instruments in all sections had better centering ability in comparison with Race and Medin instruments, it seems these differences are due to different instrumentation techniques. Race and Medin systems instrument the canals with crown down technique but Mtwo prepare the canals with step back technique. These results are in accordance with Veltri (24) and Schafer (25).

The amount of dentin removal by Mtwo instruments at coronal and middle sections was significantly less than other groups, but at apical section there was no significant difference between three groups. These differences again maybe due to different instrumentation technique between Mtwo and the two other groups which is confirmed by Yun (21) and Schirrmeister (26).

Further studies which evaluate cleaning effectiveness and instrument deformation are suggested.

## CONCLUSION

According to the results of this study Mtwo instruments had better centering ability and removed dentin more conservatively, at coronal and middle portions, than Race and Medin instruments.
